# Global Analysis of miRNA Signature Differentially Expressed in Insulin-resistant Human Hepatocellular Carcinoma Cell Line

**DOI:** 10.7150/ijms.41999

**Published:** 2020-02-24

**Authors:** Linjing Li, Yan Cheng, Li Lin, Zhuan Liu, Shengfang Du, Li Ma, Jing Li, Zhiheng Peng, Jing Yan

**Affiliations:** 1 Department of Clinical Laboratory Center, The Second Hospital of Lanzhou University, Lanzhou 730000, P.R. China.; 2 Northwest University for Nationalities, Lanzhou 730000, P.R. China.; 3 Hematology Department, Gansu Provincial Cancer Hospital, Lanzhou, Gansu 730000, China.; 4 Department of Anesthesiology, the Second Hospital of Lanzhou University, Lanzhou 730000, P.R. China.

**Keywords:** insulin resistance, HCC, miRNA Signature

## Abstract

Chemoresistance mediated by insulin resistance (IR) in HCC has already been validated. However, the underlying mechanism, especially the involvement of microRNAs (miRNAs) was unelucidated. In this study, miRNA microarrays and bioinformatics methods were employed to determine the dysregulation of miRNA by IR in HCC cells, and quantitative RT-PCR (qRT-PCR) was applied to valid the miRNA array data. Of all the 2006 miRNAs screened, 32 miRNAs were found up or down regulated between the HepG2/IR cells and its parental cells. Further literature mining revealed that some of these miRNAs may function as oncogenes or tumor suppressors that contribute to tumor progression, recurrence, and metastasis which eventually lead to chemotherapeutic resistance. Interestingly, bioinformatics analysis by Gene Ontology (GO) enrichment pathway indicating that function of the predicted target genes of these dysregulated miRNAs were significantly enriched in the processes related with biosynthesis, catabolism, modification etc., and Kyoto Encyclopedia of Genes and Genomes (KEGG) mapping showed that the biological regulatory mechanisms were integrated in cancer-related pathways. Moreover, we also constructed a network which connected the differentially expressed miRNAs to target genes, GO enrichments and KEGG pathways to reveal the hub miRNAs, genes and pathways. Collectively, our present study demonstrated the possible miRNAs and predicted target genes involving in the pathophysiology of insulin resistant HCC, providing novel insights into the molecular mechanisms of multidrug resistance in the insulin resistant HepG2 cells.

## Introduction

Hepatocellular carcinoma (HCC) is one of the most common cancers with high mortality rates worldwide 1. Unfortunately, some HCC patients are not eligible for radiofrequency ablation or surgical resections, thus must only rely on chemotherapy. However, the inherent multidrug resistance (MDR) towards chemotherapeutics often impairs the effect of chemotherapy for HCC therefore cannot produce satisfactory clinical outcomes 2. Drug resistance is a multifactorial phenomenon involving many mechanisms, such as T2DM and IR caused by tumorigenesis and inflammation in the liver 3-5. Our previous studies also validated that IR enhances the tolerance of HCC cells to chemotherapeutic 6-9, indicating that IR contributes to the inherent MDR of HCC. Nevertheless, the key underlying mechanism of the acquisition of resistance to chemotherapeutic drugs still remain largely unexplored.

miRNAs are endogenously expressed small non-coding RNAs with 18-25 nucleotides which play a vital role in the regulation of gene expression at post-transcriptional level 10. Emerging evidences have revealed that miRNAs are important modulators in many signaling pathways involved in tumorigenesis 11. Furthermore, significant variation with miRNA expression were observed in drug tolerant cancer cells in comparison with their parental drug sensitive cancer cells which displays as higher expression of cancer-promoting miRNAs (oncomiRNAs) and lower expression of cancer-inhibiting miRNAs (tumor suppressor miRNAs) 12.They might serve as tumor suppressors or oncogenes and constitute ideal targets in exploring anticancer therapeutics 13. miRNAs have been showed as regulators that can promote or impair drug resistance in several cancers including HCC. Kabir TD et al reported that miR-7 could overcome sorafenib resistance by suppressing its target TYRO3 via PI3-Kinase/AKT pathway 14. Xia H etc. declared that miR-216a/217 could activate TGF-β pathway to induce sorafenib resistance 15. Meng W etc. believed that inhibition of the expression of miR-33a-5p could reduce cisplatin sensitivity and increased its drug resistance in HCC 16. Another group identified the role of miR-195 in developing drug resistance in HCC cell line 17. They found that it might improve 5-FU sensitivity by targeting Bcl-w protein to increase cell apoptosis. It was also reported that miR-3129-5p and its target gene Zeb1 endow HCC cells a tolerance to doxorubicin 18. Due to the important role of miRNAs in the development of drug resistance of HCC, it is useful to employed global and systematic analytic techniques to assess the miRNA expression profiles in the drug resistant HCC cells.

Chemoresistance mediated by insulin resistance in HCC have already been validated by our previous studies 6-9, while little was known concerning the role of miRNAs in IR-mediated chemotherapy resistance. Thus, in the current study, comprehensive expression profiling of miRNAs by microarray was performed in insulin-resistant and parental HCC cell lines. Differentially expressed miRNA were identified between these two cell lines with bioinformatics analyses, which may contribute to a better understanding of the potential role of miRNAs in multidrug resistance of the insulin-resistant HCC cells.

## Materials and Methods

### Cell culture

Human hepatocellular carcinoma HepG2 cells were purchased from American Type Culture Collection (ATCC HB-8065, Rockville, MD, USA) and cultured in Dulbecco's modified eagle medium (DMEM) supplemented with 10% FBS at 37 °C with 5% CO_2_. IR was induced in HepG2 cells according to the method described previously 7. Briefly, cells were incubated in serum-free DMEM for 6 hr to synchronization then treated with insulin at a concentration of 0.5 μM for 72 hr. The resultant cells were named as HepG2/IR cells.

### RNA extraction

Total RNAs from each sample were individually isolated using QIAzol Lysis Reagent and miRNeasy mini kit (Qiagen Inc, Valencia, CA, USA) according to the manufacturers' instructions. This procedure efficiently recovered all types of RNAs, including miRNAs. RNA quantity and quality were measured using a NanoDrop spectrophotometer (ND-1000; Nanodrop Technologies, Wilmington, DE, USA) and RNA integrity was detected by gel electrophoresis.

### Affymetrix GeneChip miRNA Array Hybridization

For analysis with Affymetrix GeneChip miRNA Arrays (Affymetrix, Santa Clara, CA, USA), each sample was prepared with 150 ng of total RNAs. Samples were labeled with the FlashTag Biotin HSR labeling kit (Affymetrix, Santa Clara, CA, USA) according to the manufacturer's instructions. The RNA sample was mixed into the Poly A Tailing master mix, and FlashTag™ Biotin HSR Ligation was performed by adding FlashTag Biotin HSR Ligation Mix to each of the tailed RNA samples. T4 DNA Ligase was added to each sample for labeling reaction. The hybridization cocktail was then added to each labeled sample, the resultant mix of each sample was applied to an array on the Affymetrix GeneChip miRNA 4.0 Arrays, the probe arrays were washed and stained, then scanned and analyzed using the Affymetrix GeneChip Fluidics Station 450. Differentially expressed miRNAs were identified using fold changes as well as P values calculated by the Student's t-test. The thresholds set for up and down regulated miRNAs were a fold change ≥2.0 and P≤0.05.

### Bioinformatics analysis

Potential target genes of differentially expressed miRNAs were predicted using DIANA-miRPath online software 19, with Micro T threshold set at 0.8 and P-value threshold set at 0.05. The predicted target genes subsequently underwent Gene Ontology (GO) analysis for functional annotation analysis and Kyoto Encyclopedia of Genes and Genomes (KEGG) database analysis to identify the enriched functions and pathways that might be involved. Fisher's exact and Chi-square tests were used to determine the significance of the GO and KEGG terms and pathways. Only the KEGG terms with a P-value<0.01 and GO terms with a P-value<1E-10 were selected.

### miRNA-gene network analyses

To improve the understanding of the associations between the differentially expressed miRNAs and its target genes, miRNA-gene network was constructed according to the regulatory associations between miRNAs and target genes. The associations of the genes and miRNAs were constructed by Cytoscape 3.7.2 software. The output degree was used to measure the regulated effect of miRNAs on genes or effect of genes on miRNAs.

### miRNA-GO network analysis

The miRNA-GO network was constructed according to the significantly expressed miRNAs and the results of the GO analysis. The degree of miRNAs indicated the number of GOs which were regulated by the miRNA in the network. In the same way, a higher degree of GO indicated that more miRNAs involved in the GO category. Similarly, a higher degree of miRNA suggested more GO categories related with certain miRNA.

### miRNA-KEGG network analysis

A miRNA-KEGG network was constructed according to the specifically regulated miRNAs and the KEGG analysis. The degree of miRNAs indicated the number of KEGG pathways which was regulated by the miRNA in the network. In the same way, a higher degree of KEGG indicated that more miRNAs involved in the KEGG pathway.

### Quantitative reverse transcription polymerase chain reaction (qRT-PCR)

To validate the authenticity of miRNA expression detected by microarray assay and the crucial predicted target genes of these miRNAs, 14 differentially expressed miRNAs were analyzed by qRT-PCR, including down-regulated miRNAs such: as hsa-miR-134-5p, hsa-miR-5195-3p, hsa-miR-641, hsa-miR-3935 and; up-regulated miRNAs such as hsa-miR-1231 (ID: hsmq-0529), hsa-miR-6726-5p (ID: hsmq-2842), has-miR-3180 (ID: hsmq-1188), sa-miR-3613-5p (ID: hsmq-1112), hsa-miR-6779-5p (ID: hsmq-2840), hsa-miR-7111-5p (ID: hsmq-2216), hsa-miR-6870-5p (ID: hsmq-2604), hsa-miR-6813-5p (ID: hsmq-2845), hsa-miR-4492 (ID: hsmq-1344) and hsa-miR-4505 (ID: hsmq-1358). For this analysis, the miRNAs were detected using the All-in-One™ miRNA qRT-PCR Detection Kit (GeneCopoeia, Rockville, MD, USA) according to the manufacturer's instructions. Item ID of each primer is showed before. qPCR was performed using SYBR-Green (Thermo Fisher Scientific, Inc.) reagent according to the manufacturer's instructions in the ABI 7300 real-time qPCR system (Thermo Fisher Scientific, Waltham, MA, USA). The relative abundance of each miRNA was calculated by the comparative Ct method (2-ΔΔCt), and the results were assessed by using a t-test.

### Statistical analysis of qRT-PCR result

All quantitative data were presented as means ± standard deviations. Comparisons between HepG2/IR and it parental cells were performed using the Student's t-test. P-values <0.05 were considered statistically significant. Statistical values were calculated using SPSS software, version 20.0 (IBM Corp, Armonk, NY, USA).

## Results

### miRNA microarray analysis

**Figure 1:**
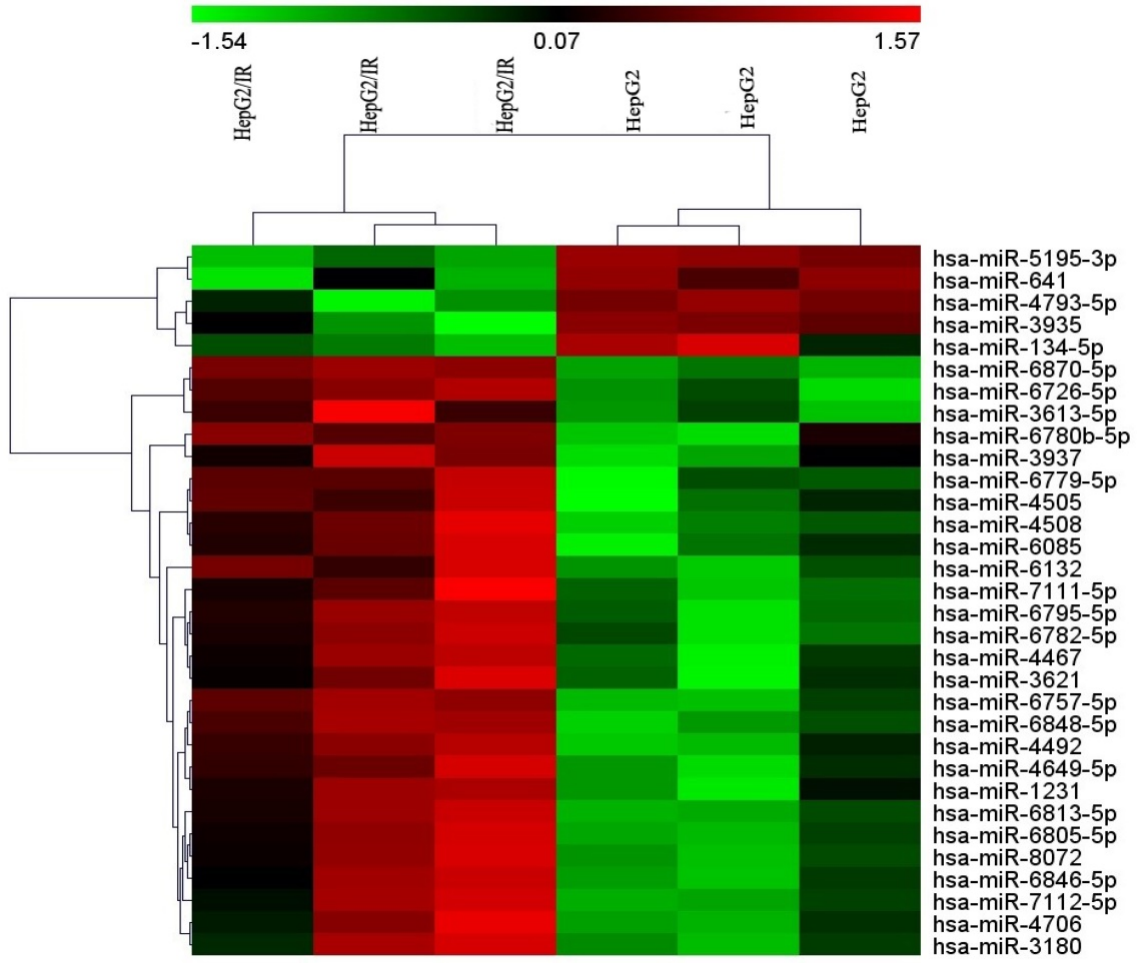
Heatmap of the microarray hybridization result showed the differentially expressed miRNAs between HepG2/IR cells and its parental cells (HepG2 cells). Total RNA was extracted from both of two cell lines, miRNAs microarrays were performed as described in Materials and Methods, both cell lines were tested in triplicate.

**Figure 2:**
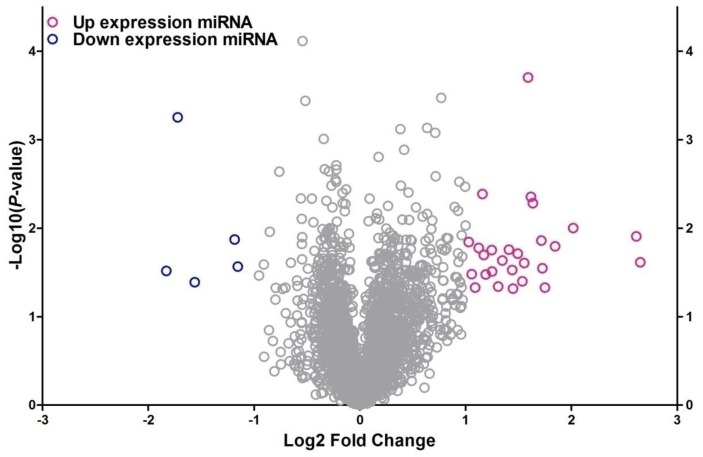
** Volcano plot of differentially expressed miRNA between HepG2/IR and HepG2 cells.** The x-axis shows the Log2 fold-change in miRNA expression and y-axis shows the -Log10 of the p-value from HepG2/IR versus HepG2 cells. Labelled miRNAs have Log2 fold change greater than 1SD from mean.

To identify the differentially expressed miRNAs between HepG2/IR cells and its parental cells, both cell lines were subjected to the Affymetrix microRNA 4.0 array in triplicate. The results revealed that, of all the 2006 miRNAs screened, 32 miRNAs were dysregulated in HepG2/IR cells compared with its normal control (Table 1) under the condition of 'fold change>2 and P<0.05'. Among these 32 miRNAs, 27 were up-regulated and 5 were down-regulated. An unsupervised 2D‑cluster analysis was applied for these two cell lines (Fig. 1) Volcano plot of significantly expressed miRNAs between HepG2/IR and HepG2 cells were showed in Fig. 2 and the 32 of them were highlighted.

### Target genes and bioinformatics analysis

**Figure 3:**
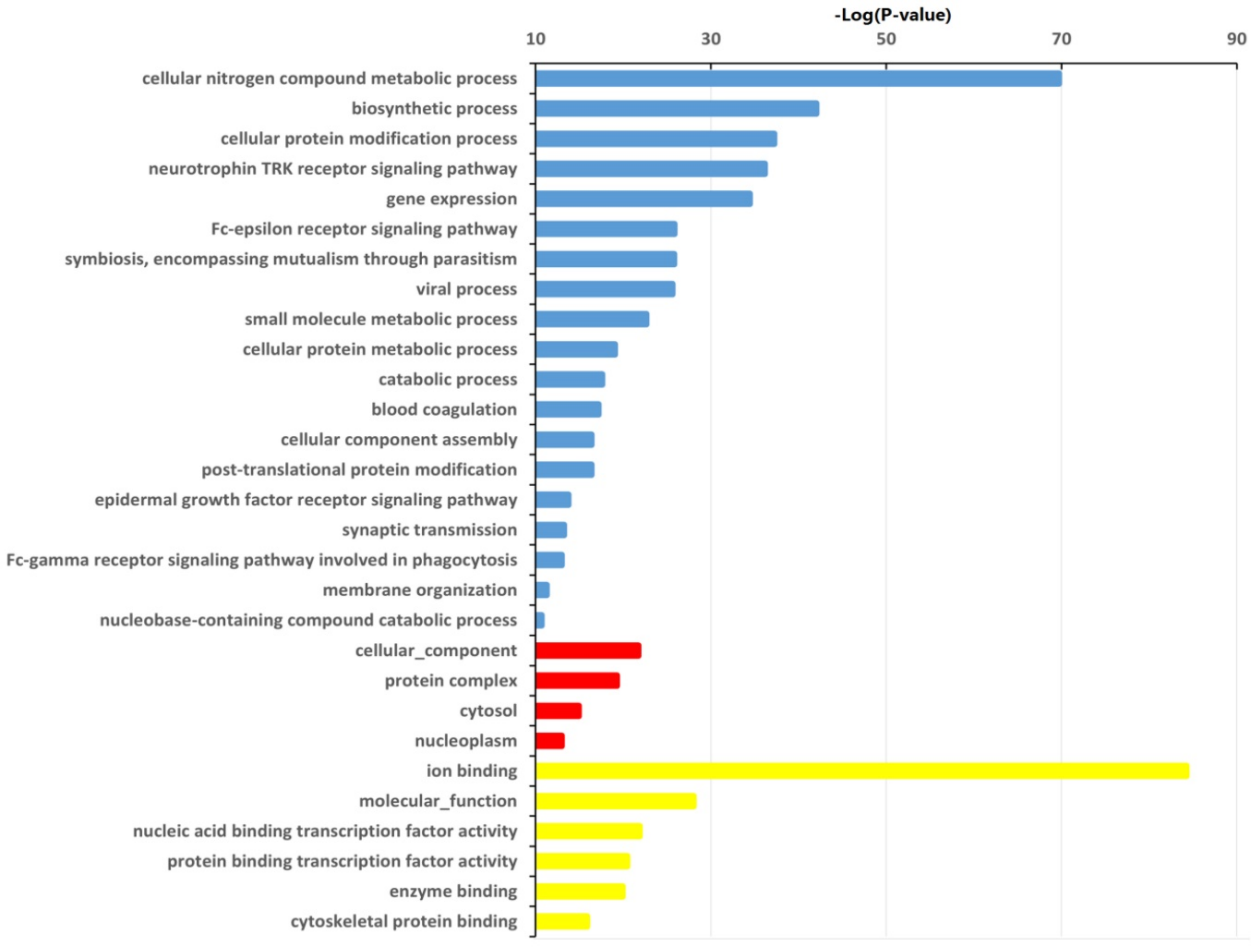
** Significantly changed GO categories of predicted target genes of deregulated miRNAs between HepG2/IR and HepG2 cells.** The y-axis shows GO category and the x-axis shows -lg*P*. The larger -lg*P* indicated a smaller *P* value. -lg*P* represent the negative logarithm of the *P* value. Blue bars indicate biological process (BP), red bars indicate cellular component (CC) and yellow bars indicate molecular function (MF).

A total of 5,406 genes were predicted as target genes of the identified 32 differentially expressed miRNAs (Table S1). To better understand the potential implications of these dysregulated miRNAs, the target genes were subjected to GO analysis to evaluate their potential functions using DIANA-miRPath v3.0 online software (http://snf-515788.vm.okeanos.grnet.gr/). In the present study, the top GO terms of the target genes of the dysregulated miRNAs were regulation of cellular nitrogen compound metabolic process, biosynthetic process, cellular protein modification process (BP); cellular component, protein complex, cytosol (CC); ion binding, transcription coactivator activity, molecular function, nucleic acid binding transcription factor activity (MF) (Fig. 3, Table S2).

**Figure 4:**
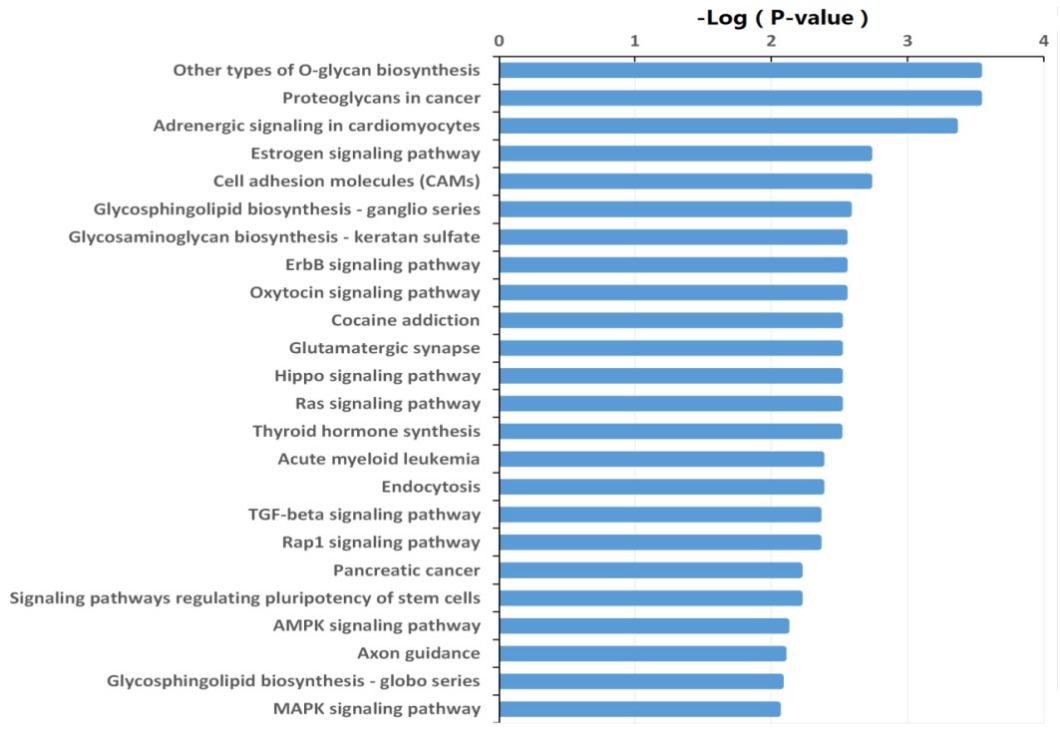
** Significantly changed KEGG pathways of predicted target genes of deregulated miRNAs between HepG2/IR and HepG2 cells.** The y-axis shows KEGG category and the x-axis shows -lg*P*. The larger -lg*P* indicated a smaller *P* value. -lg*P* represent the negative logarithm of the *P* value.

The target genes were also subjected to KEGG pathway enrichment analysis using DIANA-miRPath v3.0 online software (http://snf-515788.vm.okeanos.grnet.gr/) to determine the canonical pathways controlled by the identified miRNAs. Other types of O-glycan biosynthesis, proteoglycans in cancer, adrenergic signaling in cardiomyocytes, estrogen signaling pathway, cell adhesion molecules, glycosphingolipid biosynthesis, glycosaminoglycan biosynthesis, and ErbB signaling pathway were the most active pathways that the target genes of the differentially expressed miRNAs may be involved. (Fig. 4, Table S3).

### miRNA-gene-network analyses

**Figure 5:**
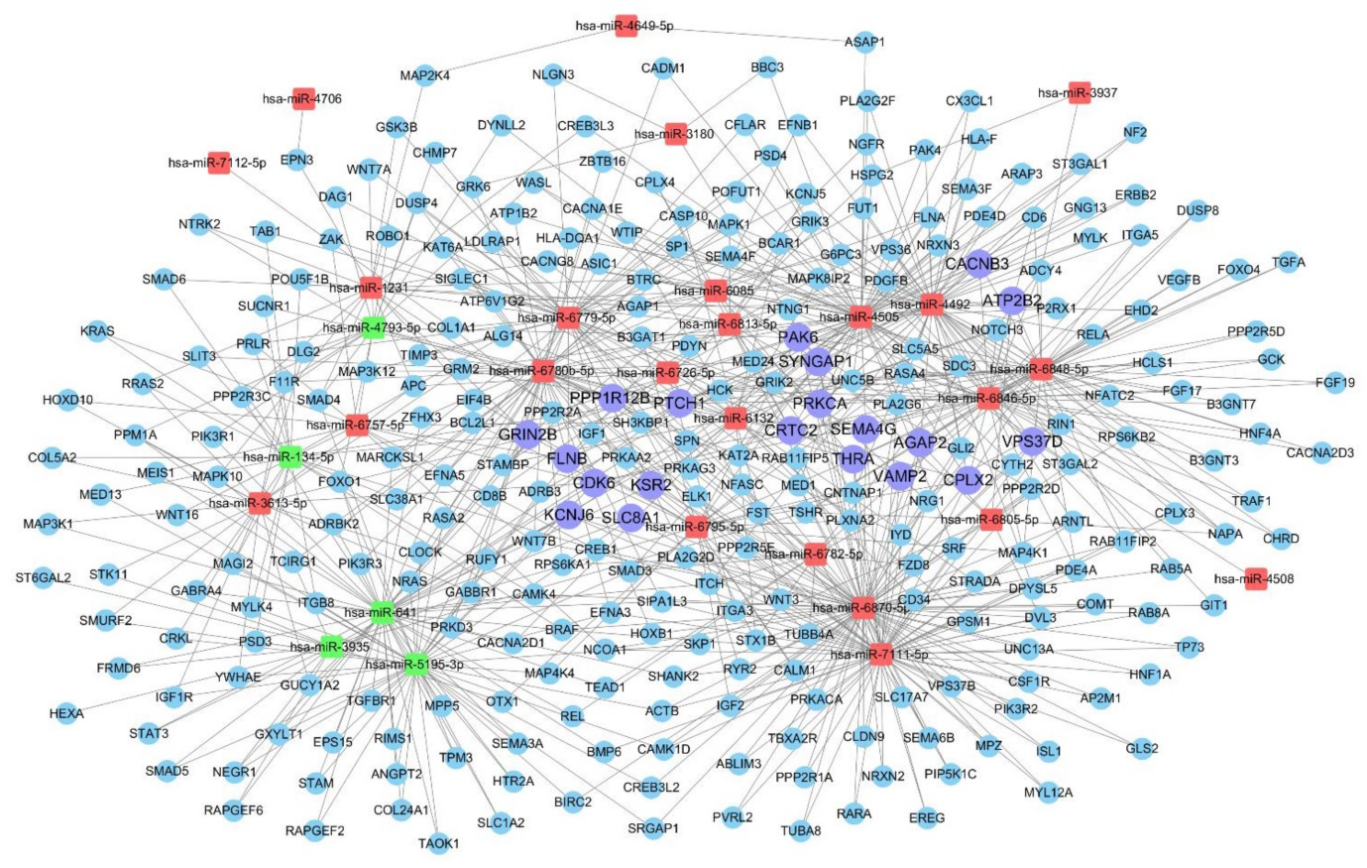
** miRNA-gene network showing the interactions between key miRNAs and the predicted hub genes.** The square nodes represent miRNAs (red nodes denote up-regulated miRNAs, green nodes denote down-regulated miRNAs), circular nodes represent hub target genes (purple nodes denote crucial and hub target genes with degree >4).

A miRNA-gene network was constructed according to the results of the GO and KEGG pathway analyses. The core miRNAs of the interaction network include miR-6870-5p, miR-7111-5p, miR-4505, miR-4492 and miR-641 (Table 2). The network also revealed that Kinase suppressor of Ras 2 (KSR2), Ras/Rap GTPase-activating protein SynGAP (SYNGAP1), Glutamate receptor ionotropic (GRIN2B), G protein-activated inward rectifier potassium channel 2 (KCNJ6), and Complexin-2 (CPLX2) were the most crucial target genes (Table 3, Table S1). The associations of miRNAs with genes were shown in Fig. 5.

### miRNA-GO network analysis

**Figure 6:**
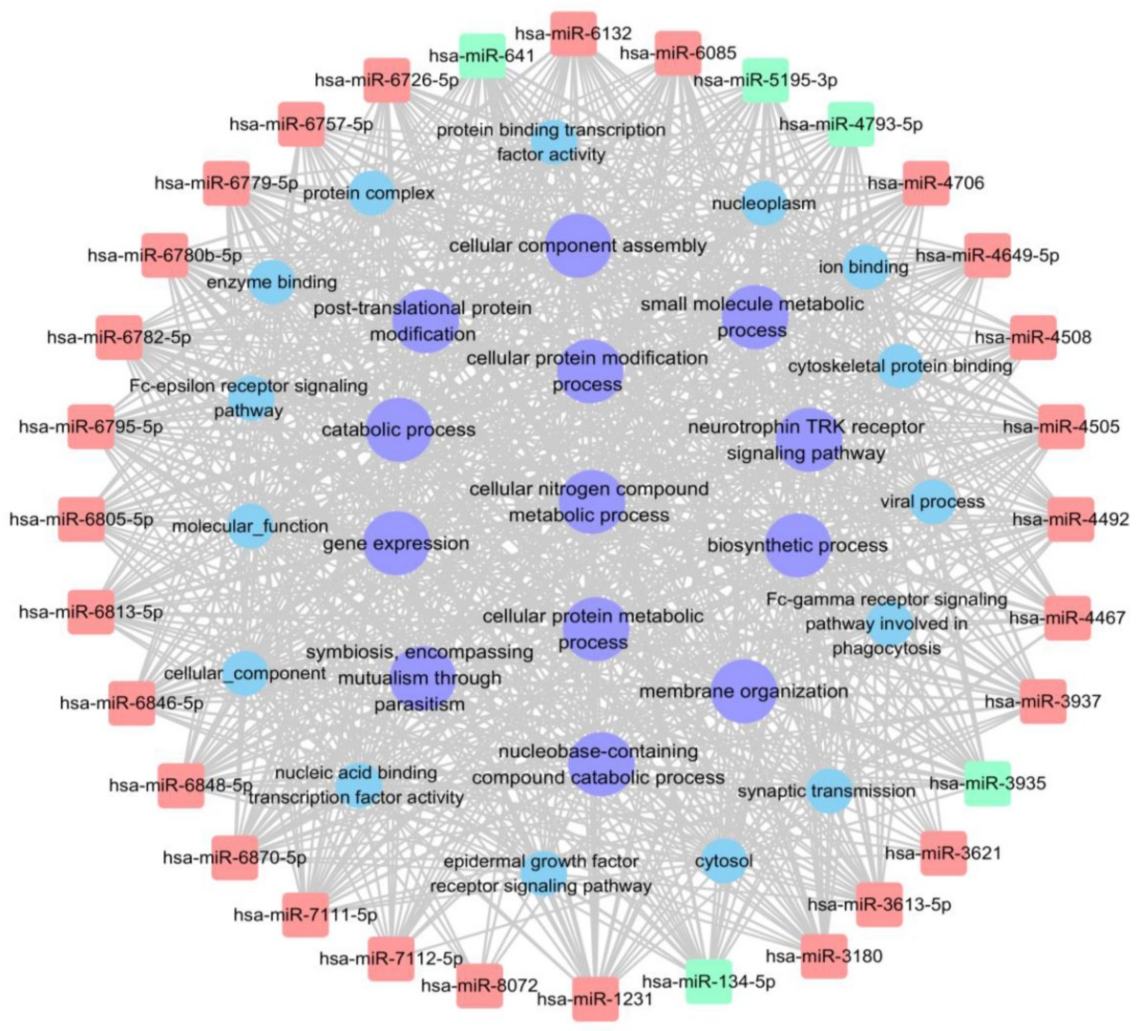
** miRNA-GO network showing the interactions between key miRNAs and the hub GO.** The square nodes represent miRNAs (red nodes denote up-regulated miRNAs, green nodes denote down-regulated miRNAs), circular nodes represent hub GO with degree>25 (purple nodes denote tumor related hub GO).

The *miRNA-*GO network analysis was favourable for determining regulatory associations between the key miRNAs and hub GO. In this network, miR-4492, miR-641 and miR-6779-5p, which contributed more than the other specifically expressed miRNAs exhibit 114, 114 and 113 GO functions, respectively (Table 4, Table S4). The most significantly regulated functional clusters of total categories were cellular nitrogen compound metabolic process, biosynthetic process, biological process and catabolic process (Table 5). The significantly complicated categories associations of miRNAs with degree more than 25 were shown in Fig. 6.

### miRNA-KEGG network analysis

**Figure 7:**
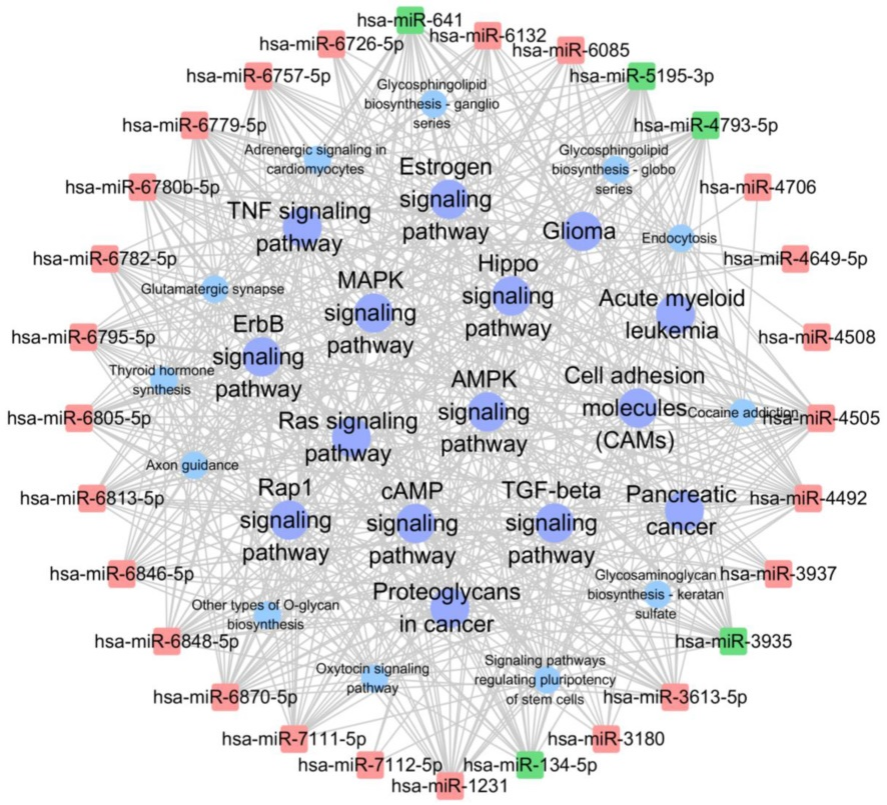
** miRNA-KEGG network showing the interactions between key miRNAs and the hub KEGG pathway.** The square nodes represent miRNAs (red nodes denote up-regulated miRNAs, green nodes denote down-regulated miRNAs), circular nodes represent hub KEGG pathway with degree>30 (purple nodes denote tumor related hub KEGG).

The network analysis was also employed to determine regulatory associations between the key miRNAs and hub KEGG pathways. In this network, miR-4492, miR-5193-3p and miR-641, which contributed more than other specifically expressed miRNAs exhibited 45, 44 and 44 KEGG pathways respectively (Table 6, Table S5). The most significantly KEGG pathway were MAPK signaling pathway, Ras signaling pathway, endocytosis and Rap1 signaling pathway, which involved in cell proliferation, growth, and differentiation (Table 7). The significantly complicated pathway associations of miRNAs with degree more than 30 are shown in Fig. 7.

### Validation of miRNA array data

**Figure 8:**
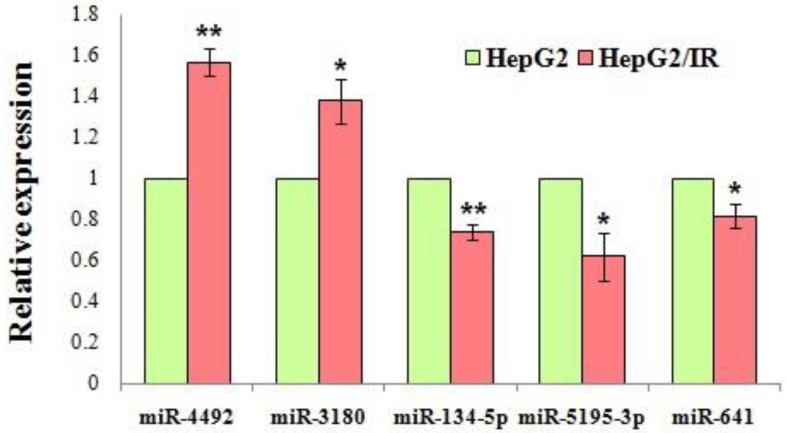
Validation of the microarray results by qRT-PCR. Expression level of the dysregulated miRNAs were shown in the figure. All miRNAs exhibited statistically significant changes in the HepG2/IR cells compared with its parental cells. Relative expression levels were calculated by 2^-ΔΔCt^ method after the (cycle threshold) Ct value (power amplification knee point) was obtained. Experiments were repeated three times with similar results, and the results are presented as mean±SD of triplicate experiments. *P<0.05, ** P<0.01.

Based on their expression levels and fold changes, 4 downregulated miRNAs (miR-134-5p, miR-5195-3p, miR-641, miR-3935) and 10 upregulated miRNAs (miR-1231, miR-6726-5p, miR-3613-5p, miR-6779-5p, miR-7111-5p, miR-6870-5p, miR-6813-5p, miR-4492, miR-4505, miR-3180) were selected for validation by qRT-PCR. The results revealed a significant difference of the expression level of miR-4492, miR-3180, miR-134-5p, miR-5195-3p, miR-641 between the IR cells and control cells, which was in a manner consistent with the data obtained from microarray analysis (P<0.05) (Fig. 8). GO enrichment analysis revealed that these validated miRNAs were significantly enriched in biosynthetic process, cellular protein modification process, gene expression, small molecule metabolic process, catabolic process, cellular component assembly, post-translational protein modification (Table S4). The pathways were also significantly enriched in the KEGG analysis, and the most involved were cancer-related pathways, including the: Ras signaling pathway, Hippo signaling pathway, ErbB signaling pathway, other types of O-glycan biosynthesis, proteoglycans in cancer and cell adhesion molecules (CAMs) (Table S5).

## Discussion

As the main anabolic hormone of the body, insulin regulates the metabolism of nutrients and promotes absorption of glucose from the blood into the cells. IR is the status when cells in the body, especially cells in the liver, fat and muscles don't respond well to insulin, and correspondingly the uptake and utilization of glucose decreases. At the whole organism level, IR will cause an impaired effect of insulin with lowering blood glucose, which may develop T2DM. To compensate for low blood glucose, the pancreas compensatory secretes excessive insulin to maintain blood sugar stability and to help glucose entering the cells. This excessive secretion leads to hyperinsulinemia in the body, which in turn activates hepatic lipogenesis and increased secretion of VLDL (hyperlipidemia) 20.

Nonalcoholic fatty liver disease (NAFLD), as one of the most common liver disorders worldwide 21, had already been reported to have tight association with IR. Although NAFLD includes a disease spectrum, ranging from simple steatosis, liver cirrhosis to hepatocellular carcinoma (HCC) etc. 22,23 and the overall occurrence from NAFLD to HCC remains a rare complication 24,25. IR had been reported to be associated with increased risk of several cancers, including HCC 26,27. It had not only been related to tumorigenesis, but also been correlated to the poor prognosis of cancer patients 3-5. Our previous studies also proved that IR could increase the invasiveness and MDR in the HCC cell line 6-9. However, the molecular mechanism of therapeutic resistance caused by IR still remains unclear.

Our present study demonstrated that 27 miRNAs were consistently upregulated and 5 miRNAs were downregulated in the HepG2/IR cells upon triplicate tests. qRT-PCR was employed to verify the expression data of 14 differentially expressed miRNAs, which showed that the expression of 5 miRNAs were in consistent with the microarray data. The outcome of qRT-PCR validated that our result of microarray screening was reliable. Among these validated miRNAs, some had already been reported to be correlated with cancer. For instance, miR-134-5p had been reported as a tumor suppressing miRNA involving in several cancers. The anti-tumor mechanisms of miR-134-5p as the potential target of various lncRNAs which function as oncogene and it can also inhibit the expression of tumor suppressor genes by directly binding to the 3'UTR region. It was confirmed that impairing the expression of miR-134-5p can promote cell proliferation, inhibit cell apoptosis, accelerate cell migration, invasion, and induce EMT and moreover contribute to multidrug resistance such as cisplatin (targeting MBTD1), 5-FU (targeting KRAS) and paclitaxel (targeting TAB1) in several carcinoma cells including non-small-cell lung, gastric, nasopharyngeal, ovarian cancers and osteosarcoma 32-35,55,56. Another microRNA reported was miR-641 which has been proved as a tumor suppressor; inhibiting proliferation, migration and invasion, as well as inducing apoptosis in lung cancer, cervical cancer and glioblastoma 36-38. It was also confirmed that miR-641 contributes to erlotinib resistance in non-small-cell lung cancer by targeting NF1 and regulating ERK signaling 39. However, little was known about the role of miR-641 in HCC. The only report about miR-641 and drug metabolism is that it acts as a direct post-transcriptional regulatory factor of CYP3A4, which is a member of the cytochrome P450 superfamily. As we all know, the cytochrome P450 proteins are monooxygenases in the hepatocytes that catalyze many reactions involved in drug metabolism, the enzyme also metabolizes some steroids and carcinogens as well 40. Until now, the functions especially in tumorigenesis and drug resistance of other validated miRNAs such as miR-3180, miR-6726-5p, miR-7111-5p etc. have remained unclear. Our previous study has reported that insulin resistant HCC cells obtain ability of multidrug resistance such as cisplatin, 5-FU, vincristine, mitomycin 7. In accordance with the researches mentioned above, miR-134-5p and miR-641 were down-regulated in insulin resistant HCC cells, thus suggesting that these known or unknown miRNAs might play a critical role in multidrug resistance of insulin resistant HCC cells especially miR-134-5p and miR-641. Further functional experiments will be conducted in our following study.

miRNA mainly performs its regulatory function through its targets. Functions of these predicted target genes significantly enriched in the processes related with metabolic, biosynthetic, transcriptional and protein modification etc., biological regulatory mechanisms were integrated in several main KEGG pathways, most of which are cancer-related pathways, such as Ras signaling pathway, Hippo signaling pathway, ErbB signaling pathway, other types of O-glycan biosynthesis etc. These pathways regulate cell proliferation, adhesion, migration, differentiation, evasion and angiogenesis 28-31. The most significant KEGG pathway was other types of O-glycan biosynthesis. It was believed that the aberrant O-glycan of cell surface influence the adhesion of cancer cells to the endothelium, promoting tumor migration, invasion and favoring cancer cells epithelial-to-mesenchymal transition (EMT). During malignancy progression, tumor cells prevalently stimulate expression of O-glycans that are normally present on embryonic tissues, but not on well-differentiated adult tissues 31,41,42. This indicates that these dysregulated miRNAs and their target genes may be involving in the tumorigenesis, drug resistance and tumor progression of the insulin resistant HCC cell line.

We also constructed a network which connected the differentially expressed miRNAs to their target genes, also to GO and KEGG enrichment. The network revealed the biological functions and contributed to better understanding of the role of dysregulated miRNAs. In these networks, certain miRNAs functioned as network hubs, such as miR-641, miR-6870-5p, miR-7111-5p, miR-4505, miR-4492, and miR-5195-3p. Among these miRNAs, miR-641 was one of the most active miRNAs in the network which targeting 52 genes, 114 GO terms and 44 KEGG pathways. In accord with previous reports, miR-641 was confirmed down-regulated in our present study, suggesting that it might be related with impaired drug sensitivity, migration and adhesion in insulin resistant HepG2 cells. Additional studies are required to elucidate the detailed role of miR-641 in insulin resistant HCC.

The miRNA networks analysis also revealed a range of hub genes (such as KSR2, SYNGAP1), crucial KEGG pathways (Ras signaling pathway, MAPK signaling pathway), and crucial GO (Cellular nitrogen compound metabolic process, Biosynthetic process, Biological process) and most of which were involved in tumorigenesis, drug resistance and poor prognosis of cancer patients 43-48. Kinase Suppressor of Ras 2 (KSR2) is a molecular scaffold that regulate the intensity and duration of the Ras/Raf/MEK/ERK/MAPK kinase cascade to facilitate energy consumption and expenditure 49,50. Deletion of KSR2 leads to impair the oxidation of fatty acids and increase their storage as triglycerides, reduced basal metabolic rate thus contributing to obesity and insulin resistance 49,51. KSR2 was also reported as an activator to stimulate tumor cell transformation 45. Ojha R et al declared that it interacts with the endoplasmic reticulum (ER) stress chaperone (GRP78) and facilitated ER translocation which drove therapy resistance in BRAF-Mutant melanoma 46. Our present study revealed that as a predicted target gene KSR2 was regulated by eight miRNAs including 7 up-regulated miRNAs (miR-4492, miR-4505, miR-6132, miR-6779-5p, miR-6780b-5p, miR-6870-5p, miR-7111-5p) and 1 down-regulated miRNA (miR-641). It was involved in many GO categories such as cellular protein modification process, cellular_component, cytosol, ion binding, molecular_function, cellular protein modification process, and participated in Ras signaling KEGG pathway. In our previous study 6-8, we found that insulin resistance contributes to multidrug resistance in HCC cells via activation of the ER stress, suggesting that KSR2 may be involved in therapy resistance in insulin resistant HCC. The expression of KSR2 and its relationship with correlated miRNAs need to be further validated.

The cytosolic protein SYNGAP1/RASA5 (SYNaptic GTPase Activating Protein 1) 48,52 encoded by SYNGAP1 gene was reported as a Ras signaling suppressor comprised in RASA subfamily (Ras GAPs) 53. It was known that Ras GAPs can inactivate RAS signaling and inhibit oncogenic transformation initiated by RAS. Suppression of Ras GAPs may constitute an additional mechanism whereby aberrant *Ras* activation promotes tumorigenesis 47,54. Li L et al 48 declared that expression of SYNGAP1/RASA5 inhibited tumor cell migration/invasion and growth in mouse model, functioning as a tumor suppressor. Conversely, knockdown of SYNGAP1/RASA5 enhanced Ras signaling to promote tumor cell growth. SYNGAP1/RASA5 also inhibited EMT through regulating actin reorganization. Thus, epigenetic inactivation of SYNGAP1/RASA5 contributing to hyperactive RAS signaling is involved in Ras-driven human oncogenesis. In our current study, SYNGAP1 was predicted being targeted only by up-regulated miRNAs including miR-4492, miR-3180, miR-4505, miR-6085, miR-6795-5p, miR-6805-5p, miR-6870-5p miR-7111-5p. It was involved in two GO categories (cellular_component and molecular_function) and participated in Ras signaling pathway. The results suggested that SYNGAP1 might be reduced expression in insulin resistant HepG2 cells. As we known, Ras signaling pathway is often deregulated in tumors through inactivation of Ras inhibitors, SYNGAP1 acts as a tumor suppressor negatively regulated the Ras signaling pathway in cancer. Decreasing expression of SYNGAP1 indicated the enhanced migration, invasion and multidrug resistance in the insulin resistant HCC. Further study should be addressed to validate the expression, regulatory miRNAs, and function of SYNGAP1 in insulin resistant HCC.

In conclusion, our study compared the miRNA expression profile in the insulin resistant HCC cells with its parental cells and identified the differentially expressed miRNAs, which provides information for further understanding of the molecular mechanisms of insulin resistance HCC cells in tumor progression and drug resistance.

## Supplementary Material

Supplementary tables.

## Figures and Tables

**Table 1:** Differentially expressed microRNAs (miRNAs) between HepG2/IR cells and its parental control (p<0.05, Fold change>2)

miRNA	p-value	Fold Change	Type of regulation
hsa-miR-3613-5p	0.0242590	6.28	Up
hsa-miR-6813-5p	0.0123839	6.12	Up
hsa-miR-6132	0.0099436	4.05	Up
hsa-miR-4492	0.0160402	3.59	Up
hsa-miR-3937	0.0468370	3.36	Up
hsa-miR-7112-5p	0.0283560	3.31	Up
hsa-miR-6795-5p	0.0137803	3.28	Up
hsa-miR-6726-5p	0.0052162	3.11	Up
hsa-miR-6848-5p	0.0044207	3.07	Up
hsa-miR-6870-5p	0.0001978	3.01	Up
hsa-miR-7111-5p	0.0247380	2.94	Up
hsa-miR-6780b-5p	0.0398410	2.90	Up
hsa-miR-6782-5p	0.0193971	2.82	Up
hsa-miR-3180	0.0482000	2.73	Up
hsa-miR-4467	0.0296810	2.72	Up
hsa-miR-6805-5p	0.0174751	2.66	Up
hsa-miR-6846-5p	0.0231760	2.54	Up
hsa-miR-4706	0.0456770	2.48	Up
hsa-miR-4505	0.0307830	2.38	Up
hsa-miR-8072	0.0176760	2.37	Up
hsa-miR-6085	0.0333000	2.28	Up
hsa-miR-4649-5p	0.0200810	2.25	Up
hsa-miR-6757-5p	0.0040993	2.23	Up
hsa-miR-6779-5p	0.0167355	2.18	Up
hsa-miR-3621	0.0468600	2.13	Up
hsa-miR-1231	0.033018	2.08	Up
hsa-miR-4508	0.0143212	2.04	Up
hsa-miR-641	0.027156	-2.23	Down
hsa-miR-4793-5p	0.0133985	-2.27	Down
hsa-miR-134-5p	0.040759	-2.95	Down
hsa-miR-5195-3p	0.00055787	-3.30	Down
hsa-miR-3935	0.030403	-3.55	Down

**Table 2:** Crucial microRNAs (miRNAs) in the miRNA-target network (degree >3)

miRNA	Degree
hsa-miR-6870-5p	87
hsa-miR-7111-5p	67
hsa-miR-4505	66
hsa-miR-4492	57
hsa-miR-641	52
hsa-miR-6780b-5p	50
hsa-miR-5195-3p	48
hsa-miR-6846-5p	46
hsa-miR-6848-5p	45
hsa-miR-6779-5p	41
hsa-miR-6795-5p	27
hsa-miR-6132	26
hsa-miR-134-5p	22
hsa-miR-3613-5p	19
hsa-miR-1231	18
hsa-miR-4793-5p	17
hsa-miR-6085	17
hsa-miR-6813-5p	16
hsa-miR-6782-5p	14
hsa-miR-3935	13
hsa-miR-6757-5p	13
hsa-miR-6726-5p	8
hsa-miR-6805-5p	8
hsa-miR-3180	4

**Table 3:** Crucial and hub target genes in the miRNA-target network (degree >4)

Crucial Target Gene	Description	Degree
CPLX2	Complexin-2	8
GRIN2B	Glutamate receptor ionotropic, NMDA 2B	8
KCNJ6	G protein-activated inward rectifier potassium channel 2	8
KSR2	Kinase suppressor of Ras 2	8
SYNGAP1	Ras/Rap GTPase-activating protein SynGAP	8
THRA	Thyroid hormone receptor alpha	8
AGAP2	Arf-GAP with GTPase, ANK repeat and PH domain-containing protein 2	7
PTCH1	Protein patched homolog 1	7
SEMA4G	Semaphorin-4G	7
VAMP2	Vesicle-associated membrane protein 2	7
CDK6	Cyclin-dependent kinase 6	6
CRTC2	CREB-regulated transcription coactivator 2	6
PAK6	Serine/threonine-protein kinase PAK 6	6
PRKCA	Protein kinase C alpha type	6
ATP2B2	Plasma membrane calcium-transporting ATPase 2	5
CACNB3	Voltage-dependent L-type calcium channel subunit beta-3	5
FLNB	Filamin-B	5
PPP1R12B	Protein phosphatase 1 regulatory subunit 12B	5
SLC8A1	Sodium/calcium exchanger 1	5
VPS37D	Vacuolar protein sorting-associated protein 37D	5

**Table 4:** Crucial miRNAs in the miRNA-GO network (degree >10)

miRNA	Degree
hsa-miR-4492	114
hsa-miR-641	114
hsa-miR-6779-5p	113
hsa-miR-6870-5p	113
hsa-miR-7111-5p	110
hsa-miR-3613-5p	109
hsa-miR-6780b-5p	108
hsa-miR-4505	106
hsa-miR-6846-5p	105
hsa-miR-6795-5p	104
hsa-miR-134-5p	101
hsa-miR-4793-5p	101
hsa-miR-6848-5p	101
hsa-miR-5195-3p	100
hsa-miR-1231	97
hsa-miR-6132	97
hsa-miR-6757-5p	97
hsa-miR-6813-5p	96
hsa-miR-6782-5p	92
hsa-miR-6085	91
hsa-miR-3935	81
hsa-miR-3937	74
hsa-miR-6805-5p	67
hsa-miR-6726-5p	63
hsa-miR-3180	59
hsa-miR-4649-5p	58
hsa-miR-4508	33
hsa-miR-4706	33
hsa-miR-7112-5p	33
hsa-miR-4467	29
hsa-miR-3621	20
hsa-miR-8072	16

**Table 5:** Crucial GO category in the microRNA-GO network (degree >20)

GO ID		GO (name)	Degree	P value
BP	GO:0034641	cellular nitrogen compound metabolic process	32	1.93E-70
BP	GO:0009058	biosynthetic process	32	8.87E-43
BP	GO:0008150	biological_process	32	2.79E-06
BP	GO:0009056	catabolic process	31	2.45E-18
BP	GO:0022607	cellular component assembly	31	4.15E-17
BP	GO:0006351	transcription, DNA-templated	31	0.0449519
BP	GO:0006464	cellular protein modification process	30	5.95E-38
BP	GO:0044281	small molecule metabolic process	30	2.21E-23
BP	GO:0006950	response to stress	30	3.49E-09
BP	GO:0034655	nucleobase-containing compound catabolic process	29	1.98E-11
BP	GO:0065003	macromolecular complex assembly	29	1.19E-10
BP	GO:0006461	protein complex assembly	29	3.48E-07
BP	GO:0016192	vesicle-mediated transport	29	0.0433165
BP	GO:0002376	immune system process	28	2.04E-06
BP	GO:0008219	cell death	28	2.97E-06
BP	GO:0006259	DNA metabolic process	28	0.0073611
BP	GO:0010467	gene expression	27	3.60E-35
BP	GO:0044403	symbiosis, encompassing mutualism through parasitism	27	1.57E-26
BP	GO:0044267	cellular protein metabolic process	27	8.96E-20
BP	GO:0048011	neurotrophin TRK receptor signaling pathway	26	6.94E-37
BP	GO:0016032	viral process	26	2.41E-26
BP	GO:0043687	post-translational protein modification	26	4.23E-17
BP	GO:0061024	membrane organization	26	5.18E-12
BP	GO:0007267	cell-cell signaling	26	1.13E-09
BP	GO:0051056	regulation of small GTPase mediated signal transduction	26	1.07E-05
BP	GO:0006367	transcription initiation from RNA polymerase II promoter	26	5.57E-05
BP	GO:0042592	homeostatic process	26	0.0079643
BP	GO:0030198	extracellular matrix organization	26	0.0361203
BP	GO:0007596	blood coagulation	25	6.77E-18
BP	GO:0007268	synaptic transmission	25	5.55E-14
BP	GO:0016070	RNA metabolic process	25	3.88E-06
BP	GO:0045087	innate immune response	25	0.0002734
BP	GO:0048870	cell motility	25	0.000421
BP	GO:0007010	cytoskeleton organization	25	0.0095913
BP	GO:0016071	mRNA metabolic process	24	3.49E-09
BP	GO:0007411	axon guidance	24	0.0001162
BP	GO:0097190	apoptotic signaling pathway	24	0.0084989
BP	GO:0007399	nervous system development	24	0.0092641
BP	GO:0006790	sulfur compound metabolic process	24	0.0392062
BP	GO:0038095	Fc-epsilon receptor signaling pathway	23	1.46E-26
BP	GO:0007173	epidermal growth factor receptor signaling pathway	23	1.72E-14
BP	GO:0030203	glycosaminoglycan metabolic process	23	2.21E-10
BP	GO:0000278	mitotic cell cycle	23	2.73E-08
BP	GO:0030168	platelet activation	23	3.73E-07
BP	GO:0008286	insulin receptor signaling pathway	23	2.61E-06
BP	GO:0019221	cytokine-mediated signaling pathway	23	0.0327518
BP	GO:0008543	fibroblast growth factor receptor signaling pathway	22	3.49E-09
BP	GO:0034142	toll-like receptor 4 signaling pathway	22	2.23E-08
BP	GO:0034330	cell junction organization	22	3.30E-06
BP	GO:0006325	chromatin organization	22	0.0046663
BP	GO:0022617	extracellular matrix disassembly	22	0.0138599
BP	GO:0006091	generation of precursor metabolites and energy	22	0.0460089
BP	GO:0038096	Fc-gamma receptor signaling pathway involved in phagocytosis	21	1.01E-13
BP	GO:0034162	toll-like receptor 9 signaling pathway	21	1.01E-09
BP	GO:0002224	toll-like receptor signaling pathway	21	6.22E-07
BP	GO:0048015	phosphatidylinositol-mediated signaling	21	1.05E-06
BP	GO:0006928	cellular component movement	21	9.77E-05
BP	GO:0006644	phospholipid metabolic process	21	0.0001544
BP	GO:0000086	G2/M transition of mitotic cell cycle	21	0.0024742
CC	GO:0005575	cellular_component	32	1.93E-22
CC	GO:0043234	protein complex	30	5.11E-20
CC	GO:0005829	cytosol	30	1.17E-15
CC	GO:0005654	nucleoplasm	30	1.03E-13
CC	GO:0005815	microtubule organizing center	26	0.0031329
MF	GO:0043167	ion binding	32	5.38E-85
MF	GO:0003674	molecular_function	32	9.58E-29
MF	GO:0001071	nucleic acid binding transcription factor activity	30	1.33E-22
MF	GO:0000988	protein binding transcription factor activity	27	3.47E-21
MF	GO:0019899	enzyme binding	27	1.21E-20
MF	GO:0008092	cytoskeletal protein binding	28	1.25E-16
MF	GO:0030234	enzyme regulator activity	29	2.03E-10
MF	GO:0022857	transmembrane transporter activity	28	9.52E-05
MF	GO:0030674	protein binding, bridging	23	0.0343038

**Table 6:** Crucial miRNAs in the miRNA-KEGG network (degree >10)

miRNA	Degree
hsa-miR-4492	45
hsa-miR-5195-3p	44
hsa-miR-641	44
hsa-miR-4505	43
hsa-miR-6870-5p	43
hsa-miR-6848-5p	42
hsa-miR-6779-5p	41
hsa-miR-134-5p	39
hsa-miR-6780b-5p	39
hsa-miR-6795-5p	39
hsa-miR-7111-5p	39
hsa-miR-1231	38
hsa-miR-6846-5p	38
hsa-miR-4793-5p	37
hsa-miR-6757-5p	37
hsa-miR-3613-5p	34
hsa-miR-3935	32
hsa-miR-6813-5p	32
hsa-miR-6782-5p	28
hsa-miR-6805-5p	28
hsa-miR-6132	27
hsa-miR-6085	21
hsa-miR-3937	15
hsa-miR-3180	14

**Table 7:** Crucial KEGGs in the miRNA-KEGG network (degree >30)

KEGG ID	KEGG_Term	Degree	P value
hsa04010	MAPK signaling pathway	97	0.008968411
hsa04014	Ras signaling pathway	87	0.003145657
hsa04144	Endocytosis	85	0.004307909
hsa04015	Rap1 signaling pathway	84	0.0045072
hsa04024	cAMP signaling pathway	76	0.035393834
hsa05205	Proteoglycans in cancer	73	0.000299322
hsa04921	Oxytocin signaling pathway	68	0.002905436
hsa04261	Adrenergic signaling in cardiomyocytes	65	0.000450147
hsa04390	Hippo signaling pathway	61	0.003145657
hsa04360	Axon guidance	56	0.008176788
hsa04550	Signaling pathways regulating pluripotency of stem cells	56	0.006210047
hsa04514	Cell adhesion molecules (CAMs)	56	0.001916405
hsa04728	Dopaminergic synapse	54	0.0194252
hsa04152	AMPK signaling pathway	53	0.00777182
hsa04611	Platelet activation	51	0.017005802
hsa04919	Thyroid hormone signaling pathway	49	0.035095754
hsa04724	Glutamatergic synapse	48	0.003145657
hsa04668	TNF signaling pathway	44	0.031542367
hsa04915	Estrogen signaling pathway	42	0.001916405
hsa04723	Retrograde endocannabinoid signaling	41	0.01974073
hsa04750	Inflammatory mediator regulation of TRP channels	41	0.01974073
hsa04670	Leukocyte transendothelial migration	40	0.044378808
hsa04012	ErbB signaling pathway	40	0.002905436
hsa04666	Fc gamma R-mediated phagocytosis	39	0.034514602
hsa05414	Dilated cardiomyopathy	35	0.044378808
hsa04540	Gap junction	34	0.029084915
hsa04720	Long-term potentiation	32	0.0110171
hsa04350	TGF-beta signaling pathway	31	0.0045072
